# Successful Management of Folliculitis Decalvans

**DOI:** 10.7759/cureus.52881

**Published:** 2024-01-24

**Authors:** Yash Kashikar, Vikrant Saoji, Bhushan Madke, Meenakshi S Chandak, Soham Meghe

**Affiliations:** 1 Dermatology, Jawaharlal Nehru Medical College, Datta Meghe Institute of Higher Education & Research (DMIHER) (Deemed University), Wardha, IND

**Keywords:** staphylococcus aureus, antibiotics therapy, oral doxycycline, scalp nodule, cicatricial alopecia

## Abstract

Folliculitis decalvans (FD) is a rare disease that causes inflammation on the scalp, leading to scarring alopecia. It commonly affects young and middle-aged men and is characterized by pustules, papules, scarring, hemorrhagic crusts, and erosions. The exact cause of FD is not fully understood, but it is believed that *Staphylococcus aureus* may play a role in its development. The condition is thought to be influenced by a combination of genetic, allergic, infectious, and immunological factors. This report describes a 20-year-old male patient who experienced painful pustules on his scalp for six months. The pustules first appeared on the occipital region and then spread to the crown. The patient was diagnosed with FD after a thorough clinical and pus culture examination. Treatment involved a month-long prescription of doxycycline (100 mg BD) and topical ozenoxacin (2%), which led to successful remission of the lesions.

## Introduction

Folliculitis decalvans (FD), often known as scarring alopecia, is one kind of primary cicatricial alopecia. This uncommon skin disorder is the root cause of about 10% of primary cicatrizing alopecia cases. Inflammatory neutrophilic infiltrates and scarring that results in papules and pustules around hair follicles indicate it [1]. It primarily affects males in their middle years and is more prevalent in those with darker skin tones [2,3]. Although the actual etiology of the illness remains uncertain, *Staphylococcus (S.) aureus* has been proposed as a possible culprit. There are other factors, such as the genetic link, since the illness is caused by cytotoxins or superantigens that bind to major histocompatibility complex (MHC) II molecules and has been seen in several families [4].

According to a recent systematic review overviewing 20 studies, including 282 patients ,the authors observed lack of high quality of evidence regarding the efficacy of FD-specific treatments. Antibiotics like clindamycin, rifampicin, doxycycline and azithromycin, tacrolimus, external beam radiation, isotretinoin, human immunoglobulin, adalimumab, infliximab, long-pulse ND:Yag, and red light photodynamic therapy have been tested, but the studies lack quality of evidence. However, a combination of clindamycin and rifampicin was found to be the most commonly used treatment in reviewed studies [5]. The present report consisted management of FD using a combination of two drugs with early response and no remission for a longer duration.

## Case presentation

**Figure 1:**
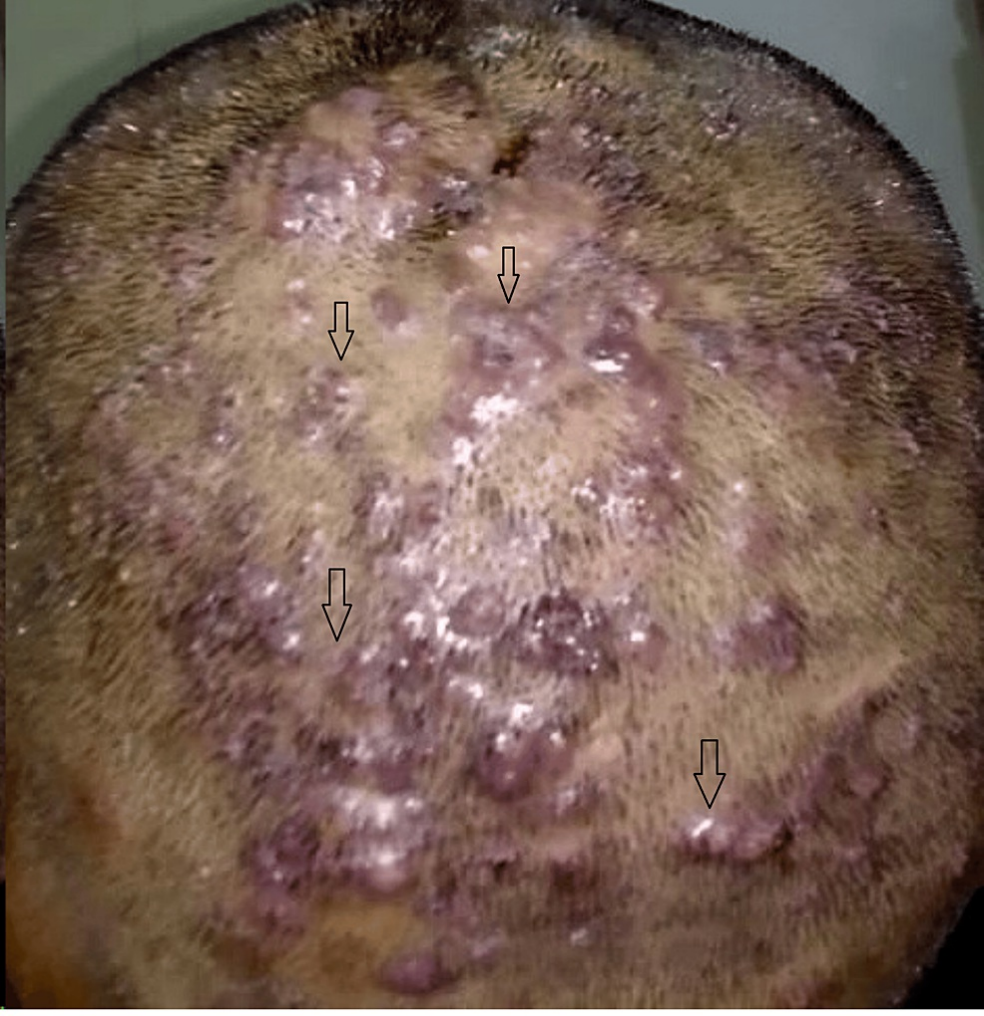
Multiple nodulocystic lesions are present on the occipital and crown area of the scalp (black arrows).

A 20-year-old male from central India presented to the outpatient department of a tertiary care hospital with multiple painful pustules covering the crown and occipital areas of his scalp (Figure 1). The patient had no history of pruritus, hidradenitis suppurativa, acne, or scalp injuries. Multiple nodulocystic lesions and patches of hair loss over the occipital and crown region were noted during the clinical examination.

**Figure 2:**
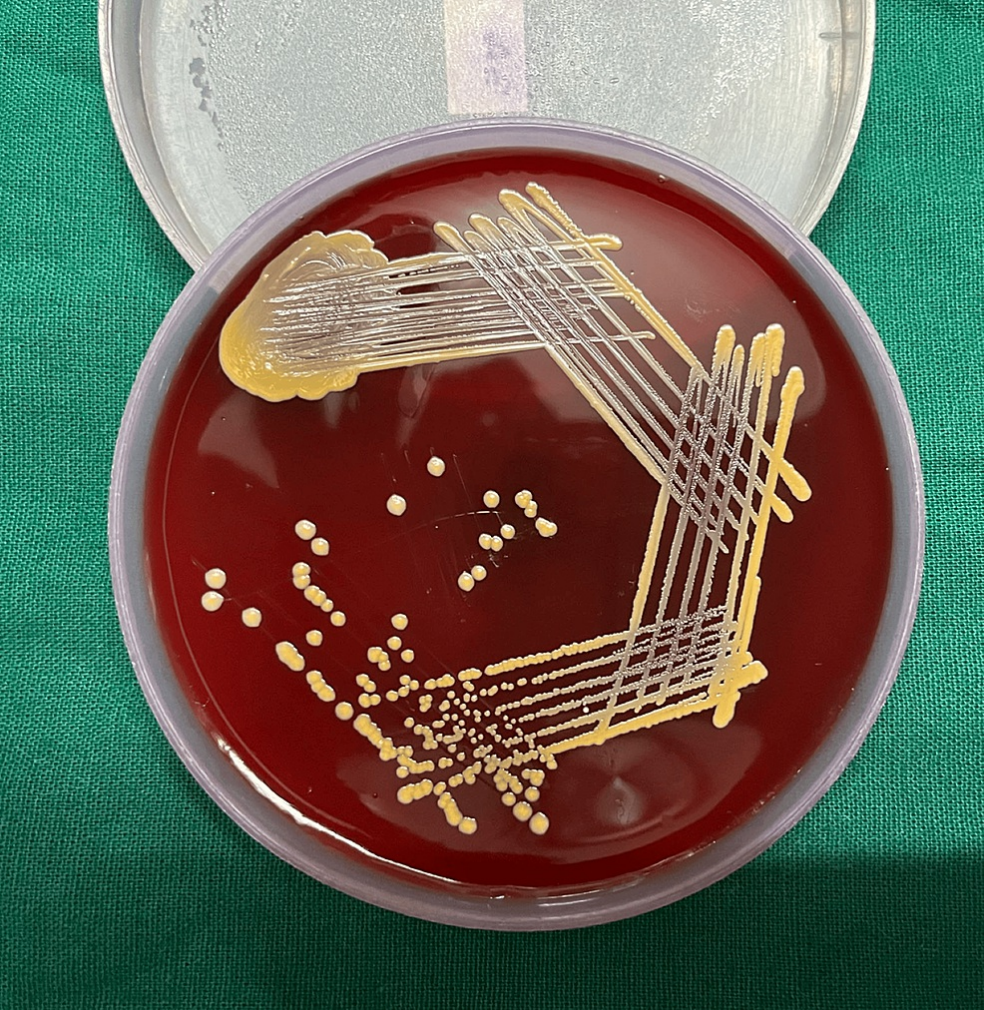
Round (1-3 mm), golden-yellow colonies of Staphylococcus aureus on blood agar.

**Figure 3:**
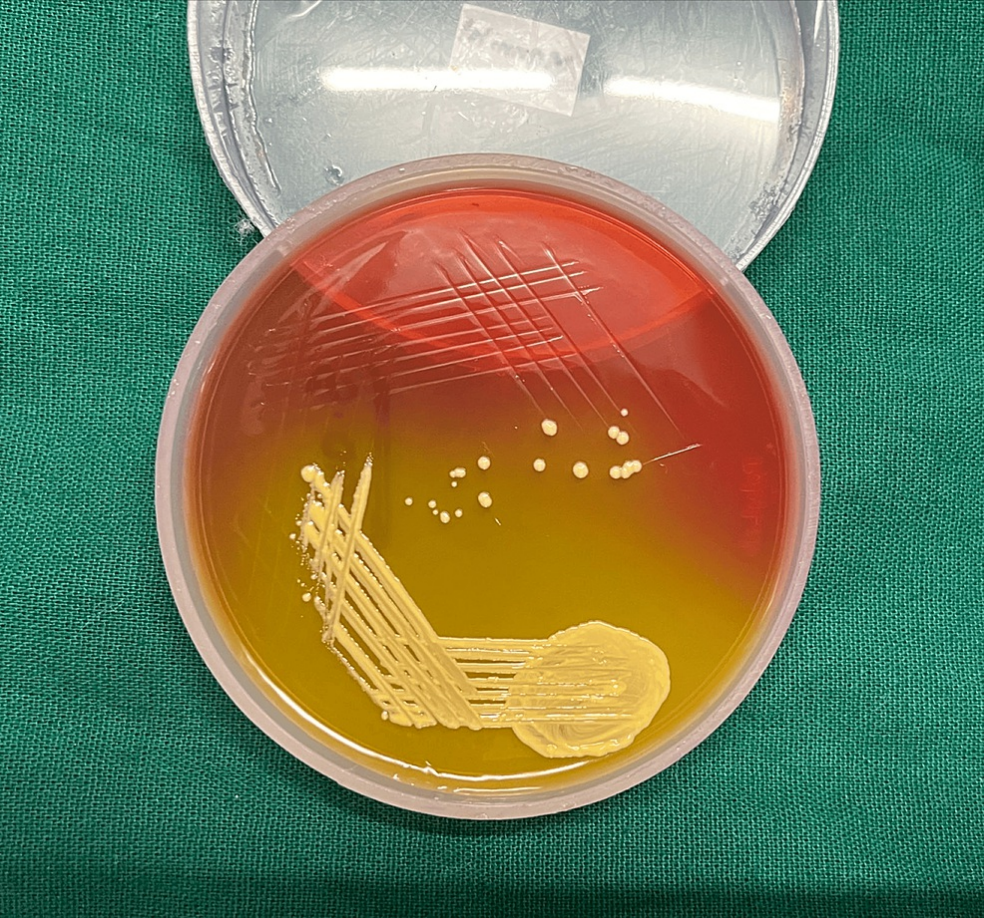
Yellow-colored colonies of Staphylococcus aureus on mannitol salt agar (MSA).

All laboratory tests, such as the fasting lipid profile, thyroid profile, liver function test, renal function test, total blood count, and other indicators of inflammation, were within normal ranges. No fungal growth was seen in the fungal culture. Methicillin-sensitive *S. aureus* was found in moderate amounts in the swabs taken from the intact pustules. Methicillin-sensitive *S. aureus *was also detected in pustule scalp bacterial cultures (Figures 2, 3).

**Figure 4:**
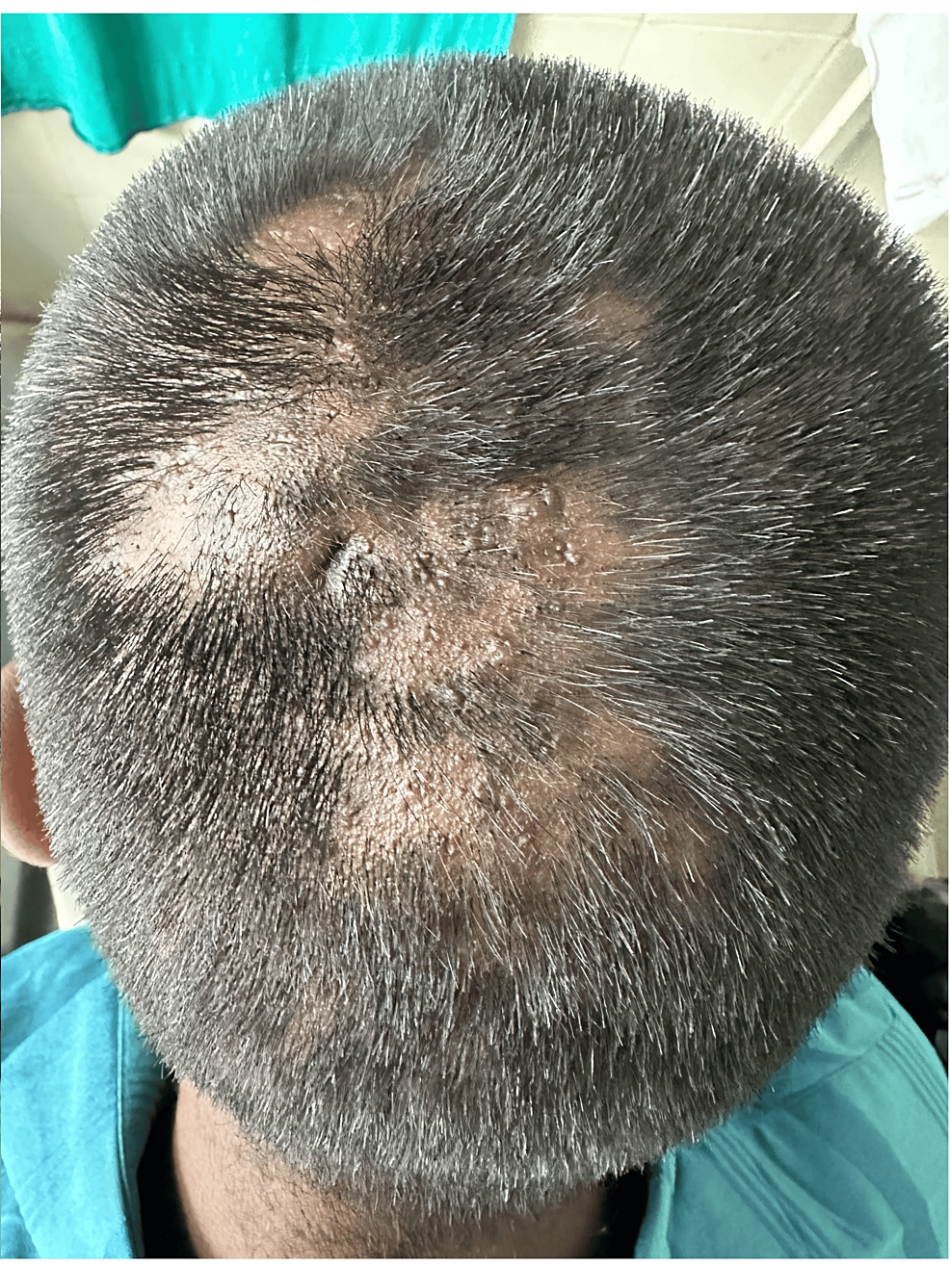
Follow-up of the patient after one month shows recession of nodulocystic lesions with well-defined reticular patches of alopecia and hair regrowth.

A diagnosis of FD was made for the patient based on the clinical presentation, examination, and culture results. The patient was prescribed topical ozenoxacin 2% lotion twice daily for one month and oral doxycycline 100 mg BD. At one-month follow-up, the patient showed a recession of nodulocystic lesions with reticular patches of alopecia and hair regrowth (Figure 4).

**Figure 5:**
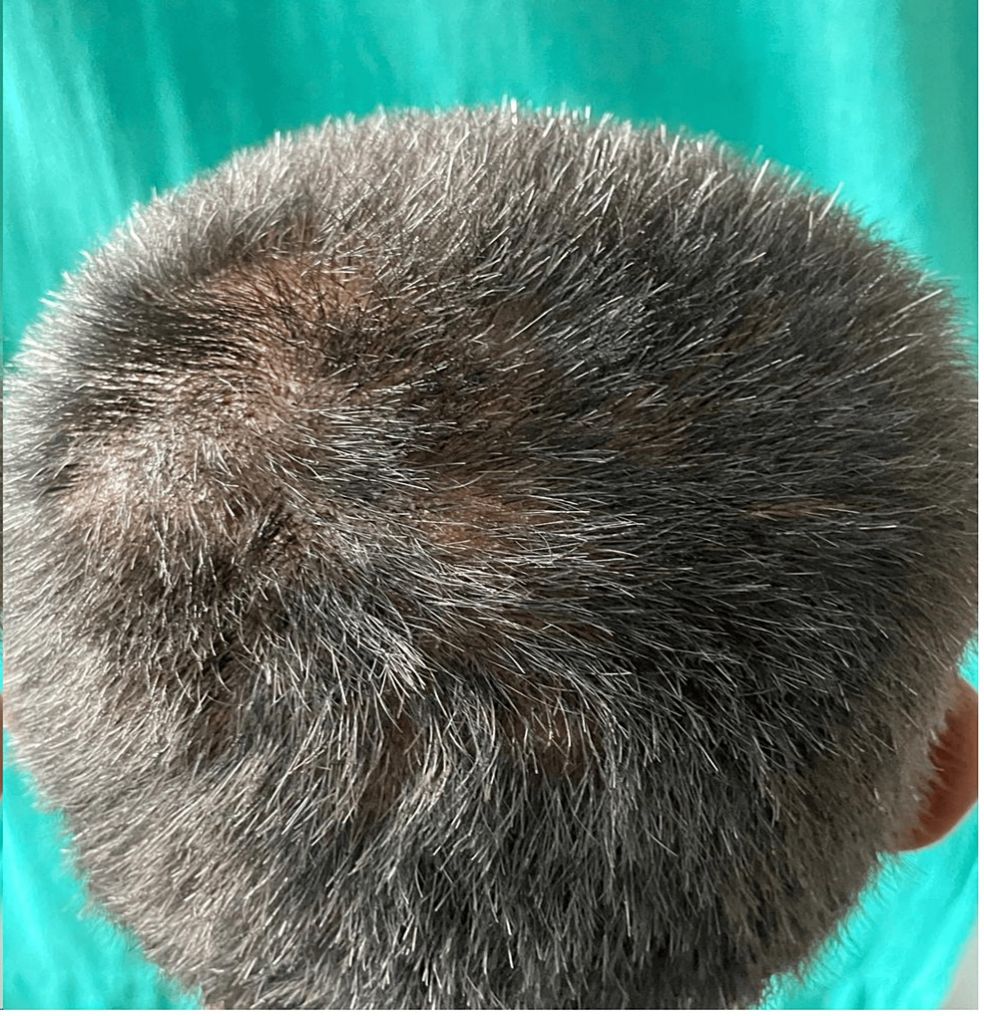
Follow-up of the patient after three months shows a complete resolution of nodulocystic lesions with hair regrowth.

There was no recurrence at the three-month follow-up with complete resolution of the nodulocystic lesions and hair regrowth (Figure 5 ).

## Discussion

FD, an unusual neutrophilic infection of the scalp, is characterized by purulent follicular exudation that is painful and recurrent, resulting in primary cicatricial baldness. The existence of superantigens and an abnormal host defense mechanism is widely accepted. However, because the etiology of FD is uncertain, therapy is difficult [1-2]. In bacterial and fungal cultures, *S. aureus *was not always the only pathogen identified; only 50% of these patients had a positive result. Although non-antibacterial therapies have also been used for FD, eliminating *S. aureus* has been the main goal as it was thought to be the only pathogen in our situation that may cause FD [2,3].

Information about the efficacy of treatments tailored to each FD is still scarce. According to the American College of Physicians Treatment Grading Guidelines, Grade 3 is the highest evidence obtained in the evidence-based evaluation study on the effectiveness of current FD treatments as revealed in a systematic review assessing the efficacy of FD treatments. According to this review, the longest disease remission lasted 7.2 months on average, while the shortest remission period was three to six months. These results were obtained with modern medication regimens that included clobetasol lotion, intralesional triamcinolone, azithromycin, clindamycin, and rifampicin [5]. It was observed that the remission period of this disease could range from two to four years. Half of the patients needed maintenance doses or treatment continuation to prevent such remission [4-6].

In the present case report, ozenoxacin and doxycycline therapies were effective, and at the six-month follow-up, there was no recurrence. The non-antibiotic (anti-inflammatory or immunomodulatory ) properties of doxycycline have been hypothesized to be the reason for its usage in the treatment of FD, which could be attributed to its inhibition of the production of proinflammatory cytokines, inhibition of proinflammatory enzymes such as inducible nitric oxide synthetase and matrix metalloproteinases, downregulation of major histocompatibility complex (MHC) class II expression in microglia and macrophages, suppression of T cell proliferation and activation, and induction of tolerogenic dendritic cells [7].

Ozenoxacin, on the other hand, is a quinolone antibiotic that stops DNA gyrase (a type II topoisomerase) and topoisomerase IV from working. Bacteria need two enzymes to make DNA [8-9]. A recent study found that ozenoxacin was more effective than levofloxacin, clindamycin, erythromycin, gentamicin, tetracycline, faropenem, and oxacillin at killing 50 different types of methicillin-susceptible *S. aureus* (MSSA), methicillin-resistant *S. aureus* (MRSA), and *Streptococcus pyogenes* bacteria [10]. Although ozenoxacin's effectiveness in treating FD has not yet been investigated, our case report indicates that the patient responded better to the combination of ozenoxacin and doxycycline. The way the two medications interact with each other may help to explain this.

## Conclusions

FD causes irreversible damage to the hair follicle. A clinician's ability to treat the patient early and effectively with minimal remission is critical to achieving its management. Since many patients are resistant to traditional therapies, it is also critical to follow up with them. In our case, the patient reacted favourably to the combination of doxycycline and ozenoxacin. Future randomized comparative trials with adequate sample size should be conducted to judge its efficacy over other management strategies.
